# Large second harmonic generation in alloyed TMDs and boron nitride nanostructures

**DOI:** 10.1038/s41598-018-27702-9

**Published:** 2018-07-04

**Authors:** Michael C. Lucking, Kory Beach, Humberto Terrones

**Affiliations:** Department of Physics, Applied Physics, and Astronomy, Rensselaer Polytechnic Institute, Troy, New York 12180 United States

## Abstract

First principles methods are used to explicitly calculate the nonlinear susceptibility (χ^(2)^(2ω, ω, ω)) representing the second harmonic generation (SHG) of two dimensional semiconducting materials, namely transition metal dichalcogenides (TMDs) and Boron Nitride (BN). It is found that alloying TMDs improves their second harmonic response, with MoTeS alloys exhibiting the highest of all hexagonal alloys at low photon energies. Moreover, careful examination of the relationship between the concentration of Se in Mo_x_Se_y_S_z_ alloys shows that the SHG intensity can be tuned by modifying the stoichiometry. In addition, materials with curvature can have large second harmonic susceptibility. Of all the calculated monolayer structures, the hypothetical TMD Haeckelites NbSSe and Nb_0.5_Ta_0.5_S_2_ exhibit the highest χ^(2)^, while one of the porous 3D structures constructed from 2D hBN exhibits a larger χ^(2)^ than known large band gap 3-D materials.

## Introduction

In the last five years, layered materials beyond graphene have attracted researchers due to the exceptional properties of their monolayer systems. The most studied have been the semiconducting transition metal dichalcogenides (STMDs) such as MoS_2_, WS_2_, MoSe_2_, and WSe_2_, whose monolayers possess a direct band gap and strong photoluminescence (PL)^1–6^, valley polarization^7–14^, strong excitonic effects^15–23^, and strong second harmonic generation (SHG)^24–33^. The case of SHG in hexagonal boron nitride (h-BN) has also been studied, but less intensively, perhaps due to the large direct band gap of around 6 eV^24,34^.

Second harmonic generation (SHG) is a nonlinear optical process in which a material interacts with an incident electric field in such a way that the frequency of incoming photons is doubled by the presence of the material^35–37^. SHG is a one of several different nonlinear optical processes that can occur in materials subject to high-energy irradiation; these include sum-frequency generation (SFG), difference-frequency generation (DFG), and optical rectification^35,36^. All of these processes emerge from higher order expansion terms of the polarization density in which the optical susceptibility tensor χ^(n)^ corresponds to the nth order set of nonlinear optical processes. Second harmonic behavior in a material is dictated by the intensity of χ^(2)^(2ω, ω, ω), a 27-component tensor, where the incoming photons ω are doubled to 2ω. Many components of χ^(2)^ usually vanish due to symmetry considerations for a given material; moreover, a potentially useful property of SHG is that any material with inversion symmetry will have identically zero second harmonic susceptibility in the dipole approximation^35^.

Although the second harmonic properties of pristine STMDs have been well studied, the effect of having alloys or curved layered phases on the SHG requires better insight. Le *et al*., have been able to synthesize MoS_2(1−x)_Se_2x_ alloys by chemical vapor deposition (CVD), finding that the SHG is more efficient in these systems^33^. In the present account we address the role of alloying monolayers by examining the SHG through first principles methods which allow the calculation of the second order susceptibility χ^(2)^. Although we do not include excitonic effects in our calculations, which play an important role in the intensities of χ^(2)^^38^, we can provide a good approximation on how the second order susceptibility behaves in alloyed TMD materials mainly due to the fact that Density Functional Theory within the local density approximation (DFT-LDA)^39^ exhibits a band gap very close to the optical band gap. In general, excitonic effects will increase the second order susceptibility^36,38,40^, thus making our calculated values an underestimate of the actual material response. Work is in progress where excitonic effects are considered. In the case of h-BN we have shifted our conduction band to the experimental optical band gap, and for the BN Schwarzites we have shifted the bands in the same proportion as the shift in h-BN. The systems we have considered are the following: different TMD monolayers including chalcogen alloys (MoSSe, WSSe, MoSTe and MoSeTe), transition metal (TM) alloys (Mo_0.5_W_0.5_S_2_, Mo_0.5_W_0.5_Se_2_) and 8–4 Haeckelites with Nb and Ta (NbSSe and Nb_0.5_Ta_0.5_S_2_), nanotube alloys (out of TMDs and h-BN), BN Haeckelites^41,42^ and BN Schwarzites which are porous BN 3-D crystals with negative Gaussian curvature^43–45^. Though some of these nanostructures have not yet been found experimentally, the results obtained could shed light on the role alloying and curvature play in nonlinear optical properties of layered materials and may motivate experimentalists to synthesize them.

Our results reveal that by alloying TMD and BN layers, the χ^(2)^ response improves in particular ranges of energy which makes them attractive for robust nonlinear optical devices. In particular, MoSTe, looks as a very promising system for SHG at lower photon energies. Interestingly, the hypothetical TMD Haeckelites (based on Nb and Ta) of the type 8–4^43^ reveal the highest χ^(2)^ of all the cases studied here. Also B_x_N_x_C_y_ Haekelites of the type 5–7^41,42^ show a very high χ^(2)^. Alloying BN layers with carbon, besides lowering the band gap, enhances the χ^(2)^ response. Another interesting result is that BN Schwarzites possess a smaller band gap than h-BN and a higher χ^(2)^ than any of the 3-D materials found so far^46^. Therefore, the presence of negative Gaussian curvature in BN enhances the nonlinear optical response. This supports the experimental finding that curvature effects can make graphene and bilayer graphene possess SHG signal^47^. Consequently, the different types of Gaussian curvature (positive or negative) play an important role in the nonlinear optical properties of the layered system.

## Results and Discussion

### Second harmonic response in semiconducting TMDs monolayers and alloys

**Figure 1:**
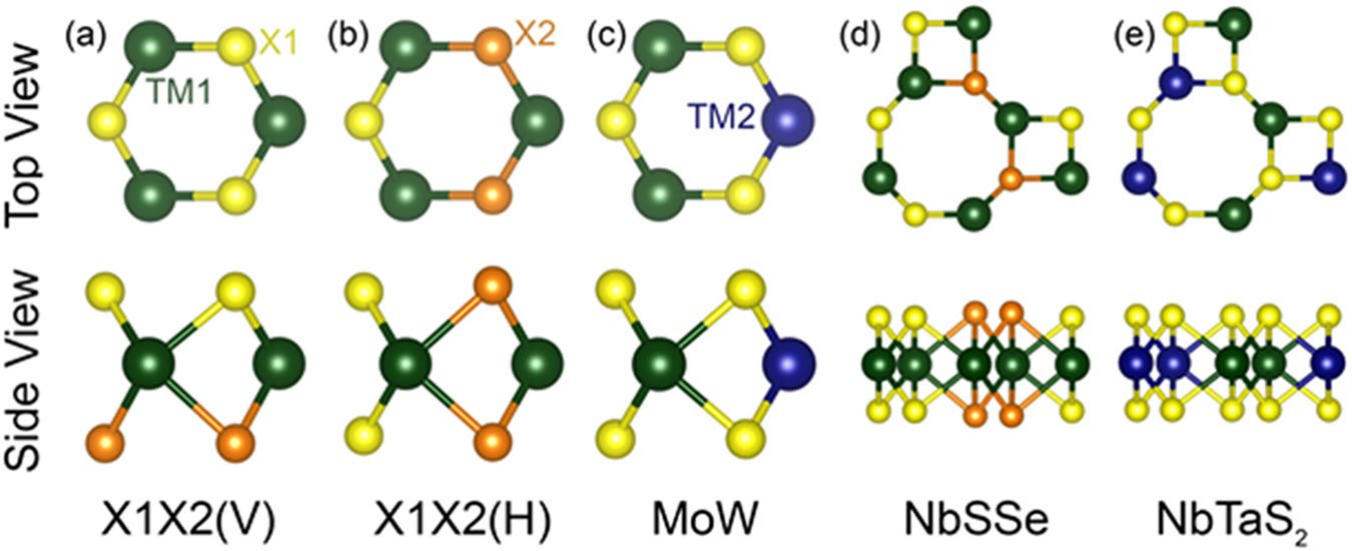
(**a**) and (**b**) Chalcogen alloys where X1X 2 can be SSe, STe or SeTe. (**c**) transition metal (TM) alloy. Haeckelite 8–4 alloys (**d**) NbSSe and (**e**) NbTaS2.

The lattice constants and band gaps of the pure and alloyed TMDs in this study are shown in Table 1. Two types of chalcogen alloys are considered: The first one, labeled MXX(V), which segregates the different chalcogens into different layers of the TMD trilayer structure (Fig. 1a). The other alloy, labeled MXX(H), separated different vertical chalcogen pairs in the in-plane direction (Fig. 1b). It is worth mentioning that recently the MoSSe(V) has been synthesized^48^. The TM alloys (Fig. 1c) are constructed in the orthorhombic cell with two MX_2_ units and have alternating “x” directional chains of the same TM atoms in the “y” direction. The resulting zigzag chains of TM atoms has been observed experimentally in CVD grown alloys^49^. The monolayer TMDs have space group number 187, P-6m2 with D_3h_ symmetry. The corresponding nonzero χ^(2)^ components are yyy = −yxx = −xxy = −xyx^35^. The (V) alloys have space group number 156, P3m1 with C_3V_ symmetry. The corresponding nonzero χ^(2)^ components are xzx = yzy, xxz = yyz, zxx = zyy, zzz, yyy = −yxx = −xxy = −xyx^35^. The (H) and TM alloys both have space group number 6, Pm with C_S_ symmetry. The corresponding nonzero χ^(2)^ components are xxx, xyy, xzz, xzx, xxz, yyz, yzy, yxy, yyx, zxx, zyy, zzz, zzx, and zxz^35^. Not surprisingly, the lattice constants of the alloys fall between the two pure materials from which they are formed. The chalcogen S and Se alloys possess optical band gaps that fall between the pure materials as expected^48^. However, the metal alloys have band gaps that are slightly lower than either of the materials that form the alloy, indicating band bowing as has been observed previously^50–52^. This is also observed for chalcogen alloys containing Te. For some cases, WSSe(V), WSe_2_, MoSe_2_, MoSSe(V), MoSeTe(V), MoSeTe(H) and MoSTe(H) the conduction band maximum (CBM) moves away from the K point, but the energy difference is small, 0.05, 0.07, 0.02, 0.002, 0.05, 0.02, 0.08 eV respectively. The MoSTe(V) alloy has a significant indirect gap which is 0.42 smaller than the direct band gap.

**Table 1:** DFT-LDA lattice parameters and band gaps of semiconducting TMDs and alloys.

TMD	Lattice Constant (Å)	LDA Band Gap (eV)
MoS_2_	3.121	1.88
WS_2_	3.129	1.99
MoSe_2_	3.245	1.62
WSe_2_	3.249	1.68
MoTe_2_	3.480	1.21
MoSSe(V)	3.183	1.75
WSSe(V)	3.188	1.83
MoSSe(H)	3.182	1.66
WSSe(H)	3.187	1.75
MoSTe(V)	3.306	1.12
MoSeTe(V)	3.364	1.39
MoSTe(H)	3.289	1.16
MoSeTe(H)	3.359	1.20
Mo_0.5_W_0.5_S_2_	3.125	1.86
Mo_0.5_W_0.5_S_2_	3.249	1.58

### Mo and chalcogen alloys

**Figure 2:**
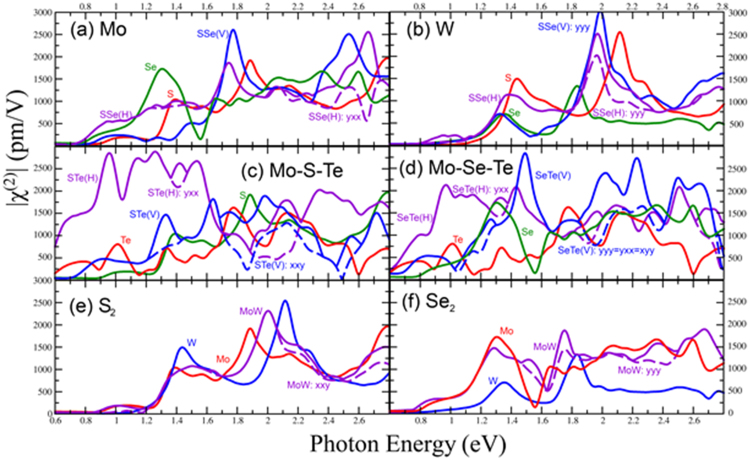
Calculated second order susceptibility of the monolayer hexagonal TMDs and alloys. Top figures show the effect of changing the chalcogen for (**a**) Mo materials and (**b**) W materials. (**c**) and (**d**) show the MoSTe and MoSeTe alloys. The upper envelope of all components of the χ^(2)^ tensor are depicted in solid lines since not only the yyy component dominates at low energies. Dashed lines are used to show the highest component of the χ^(2)^ tensor in the region below 1.5 eV. No dashed lines are used when the envelope comes from a single component. Similar case happens with WSSe(H) in figure (**b**). Figures (**e**) and (**f**) show the effect of changing the transition metal.

The calculated second harmonic response with highest susceptibility |χ^(2)^_yyy_(2ω, ω, ω)| for the MoS_2_, MoSe_2_ and MoSSe(V) are shown in Fig. 2a. For the MoSSe(H) alloy, the upper envelope of all the tensor components is shown in Fig. 2a; this is due to the fact that the symmetry of the pure trigonal prismatic is broken and other tensor components need to be considered (See Supplemental S1), therefore, by showing the envelope the main features of other components can be captured. For the MoSSe(V), the yyy component dominates all the susceptibilities and the envelope function is not necessary (See Supplemental S1). The onset in the spectra is approximately at half the optical band gap, as expected, and redshifts as one goes from S_2_ to Se_2_ (See Fig. 2a). In MoSe_2_, our spectra for low energies agrees with recent experiments with an onset at 0.8 eV and peak at 0.95 eV, which was attributed to excited excitons^53^ (See Fig. 2a and Supplemental information S2 with low smearing). There is a second peak at 1.36 eV and 1.3 eV for MoS_2_ and MoSe_2_ respectively that is absent in MoSSe(V) (See Fig. 2a). The peak at approximately 1.6 eV that has been attributed to excitonic resonance^54,55^ for MoSe_2_ appears to be slightly blueshifted in our calculation. We find that this peak comes from intraband terms, which are large in MoSe_2_ and MoS_2_. This peak is less intense than the one at 1.3 eV, but its intensity will likely increase when excitons are included in the calculation. MoSSe(V) has a large peak at 1.76 eV. The spectrum for MoSSe(H) exhibits a higher susceptibility than MoSSe(V) alloy and also larger than MoS_2_ and MoSe_2_ at low energies in the range 0.8–1.1 eV and is comparable in intensity with the MoSSe(V) at 2.6 eV, but blue shifted.

The most interesting case of the Mo chalcogen alloys involves tellurium. The χ^(2)^ for the MoSTe(H) is the highest of all hexagonal TMD alloys in the ranges between 0.8 eV and 1.6 eV photon energies (See Fig. 2c), thus we propose this alloy as a good candidate for SHG. MoSeTe also show a high χ^(2)^ in the same energy range (See Fig. 2d). For the χ^(2)^ components of the MoSTe and MoSeTe alloys see supplemental information S3.

**Figure 3:**
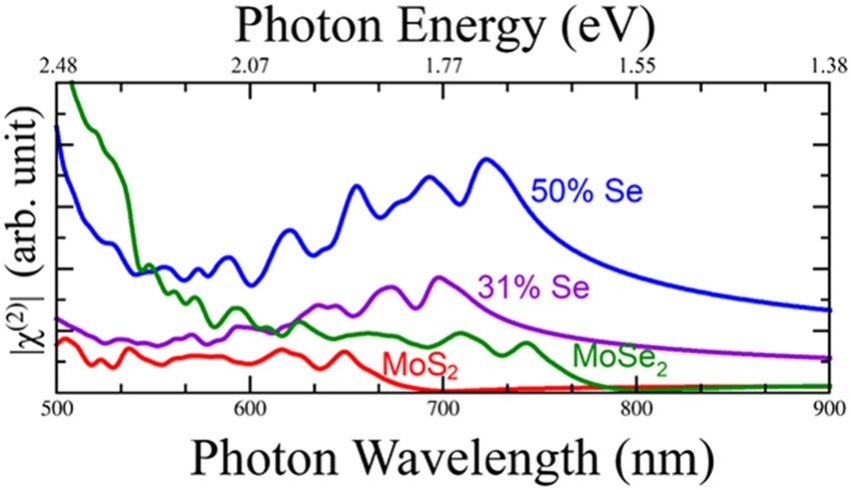
SHG of random MoS_x_Se_1−x_ alloys are compared with the two pure phases. An enhanced signal at 50% composition is in agreement with experiment^33^. The energy and wavelength correspond to the frequency doubled photon.

In order to compare our results with experimental data, MoS_x_Se_1−x_ alloys with 31% Se and 50% Se have been calculated. It is found that as Se increases the χ^(2)^ susceptibility also increases as has been demonstrated experimentally^33^ (See Fig. 3).

### W and chalcogen alloys

The second harmonic response |χ^(2)^_yyy_(2ω, ω, ω)| for the W and chalcogen alloys are shown in Fig. 2b. As in Mo chalcogen alloys, the upper envelope of WSSe(H) is shown in the figure to consider all χ^(2)^ tensor components that may play a role in the optical response (See supplemental S1). Interestingly, there is no onset at half the optical band gap for WS_2_. All other W based TMDs have a second harmonic response at half the optical gap, though not as large as is seen in the Mo based systems. WS_2_ has the largest response in the 1.4–1.6 eV range, and the peak is redshifted and the intensity is reduced with the incorporation of Se. This is at odds with previous calculations that only included the interband components which predict that WSe_2_ has a higher response^56^. Indeed our calculation shows that intraband terms dominate at this energy and are larger in WS_2_ than WSe_2._ The WSSe(V) alloy has the same peak position as WSe_2_ and its χ^(2)^ does not deviate from that of the latter appreciably at energies below 1.5 eV. At higher energies, the second harmonic response of WSSe(V) is greatly enhanced with respect to WSe_2_ and is the largest of the W based TMDs. The response of WSSe(H) is very similar to that of MSSe(H) in the sense that at energies in the range 0.8–1.1 eV possesses the highest susceptibility of all W-chalcogen alloys. One notable difference is the onset of the response at half the band gap which is absent in WS_2_.

**Figure 4:**
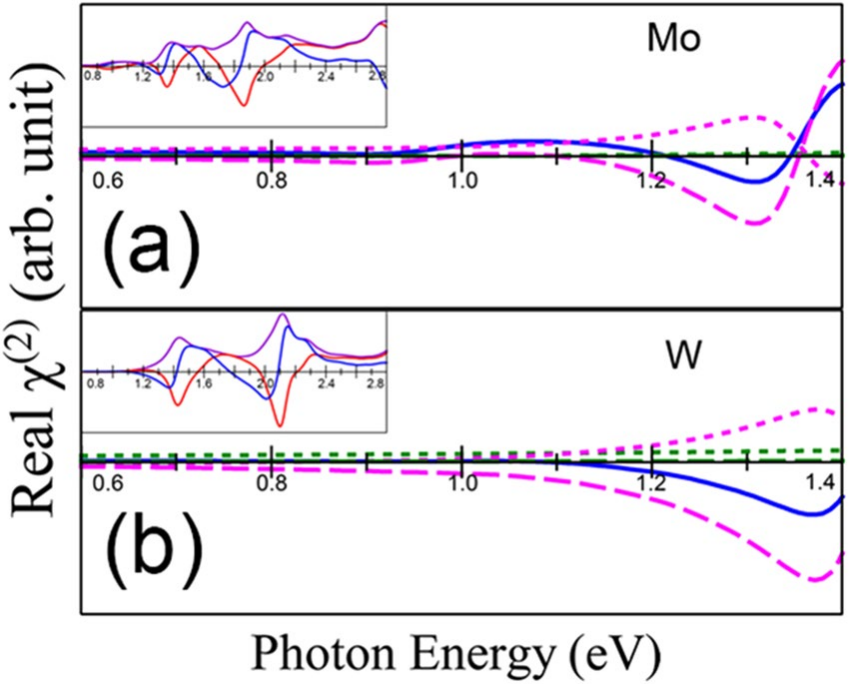
Real components of χ^(2)^ for (**a**) MoS_2_ and (**b**) WS_2_. Dotted lines are the interband terms and the dashed lines are the intraband terms. Green lines show the 1ω component and pink lines show the 2ω component. Inset: Comparison of | χ^(2)^ | (purple) with its real (blue) and imaginary (red) parts.

To shed light on the absence of a signal from WS_2_ at half the optical band gap energy, we do an in-depth comparison with MoS_2_ in Fig. 4. The origin of the second harmonic response at half the band gap energy in MoS_2_ comes from the real part of χ^(2)^, which is zero for WS_2_ (see insets in Fig. 4). The intraband 2ω term is approximately zero for MoS_2_ at half the band gap, while it has a finite negative value for WS_2_, which cancels with the positive interband term. The larger intraband term in WS_2_ signifies that the electrons at the band extrema at K are closer to the limiting case of free electrons than in MoS_2_ (See Fig. 4).

### Transition Metal Alloys

The second harmonic response of the TM alloys are shown in Fig. 2c,d. Due to the symmetry breaking and as in the case of the chalcogen alloys, the upper envelope of χ^(2)^ is shown for Mo_0.5_W_0.5_S_2_ and Mo_0.5_W_0.5_Se_2_ (See supplemental information S4 to see all the tensor components). The χ^(2)^ of Mo_0.5_W_0.5_S_2_ at half the band gap is approximately equal to that of MoS_2_, suggesting that the band extrema closely resembles this material. The peak at approximately 1.4 eV coincides with the peak for MoS_2_, but the intensity is reduced. The next peak at approximately 2 eV is halfway between the peaks for MoS_2_ and WS_2_, though the susceptibility is close to that of MoS_2_. At energies higher than 2.5 eV, the intensity of χ^(2)^ for the alloy falls between that of the two pure phases. The susceptibility of Mo_0.5_W_0.5_Se_2_ follows the that of MoSe_2_ closely up to photon energies of 1.5 eV. There is a small enhancement and redshift in the onset of the spectrum, likely due to the 0.1 eV reduction in the band gap. The peak at approximately 1.3 eV has a longer tail than MoSe_2_, but a slightly reduced intensity. The peak at 1.7 eV seems to be an average of the peaks at 1.6 and 1.8 eV for the Mo and W pure phase respectively. Above 2 eV, the susceptibility is close to that of MoSe_2_, it is not harmed by the much lower susceptibility of WSe_2_.

### TMD Haeckelites alloys

**Figure 5:**
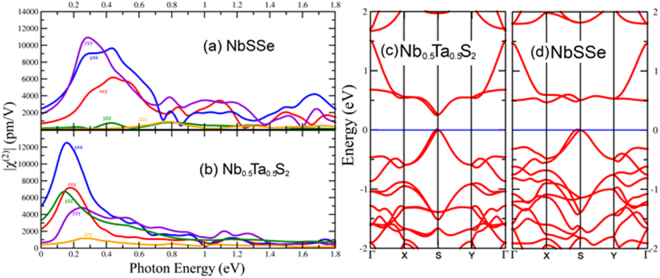
Large Second harmonic response for TMD Haeckelites (**a**) NbSSe and (**b**) Nb_0.5_Ta_0.5_S_2_. (**c**) Band structure of Nb_0.5_Ta_0.5_S_2_ Haeckelite. (**d**) Band structure of NbSSe Haeckelite.

The hypothetical TMD Haeckelites^43^ are also interesting materials. These structures are made of 8 and 4 member rings, but they contain inversion symmetry so the second order nonlinear optical response will be zero, however, alloying the Haeckelites can break the inversion symmetry to potentially give rise to a SHG response. We choose to study the Nb based Haeckelites because they possess a band gap, unlike those made from Mo or W^43^. NbSSe and Nb_0.5_Ta_0.5_S_2_ alloys were both considered (See Fig. 1d,e). The alloys are constructed such that the atoms alternate in the “x” direction but not in the “y” direction, we have alternation in the “x” direction of the “y” directional chains of similar type atoms. The Nb_0.5_Ta_0.5_S_2_ alloy has a 0.26 eV direct gap while the NbSSe alloy has an indirect gap of 0.46 eV (direct gap 0.48 eV. See Fig. 5c,d). These structures possess C_2v_ symmetry with space group Pmc2_1_ (number 26). This gives 5 independent nonzero χ^(2)^ components, zzy = zyz, xxy = xyx, yzz, yxx, and yyy^35^ (see Fig. 5a,b). The Nb_0.5_Ta_0.5_S_2_ alloy has a larger response, shown in Fig. 5b, likely due to the smaller band gap. For both alloys, the zzy component of χ^(2)^ is smallest, and the yzz is also very small for NbSSe (see Fig. 5a). The yyy component is largest for NbSSe followed closely by the yxx, while the yxx component is largest for Nb_0.5_Ta_0.5_S_2_: The peak positions are at 0.2–0.3 eV for the SHG. NbSSe exhibits a higher response at energies above 0.8 eV. The highest peak for the Nb_0.5_Ta_0.5_S_2_ alloy is nearly 13,000 pm/V, which is over 4x larger than the highest susceptibility achieved in the traditional trigonal prismatic TMD monolayered structures. Even above 0.8 eV, the NbSSe Hackelite has a SHG response that compares favorably to the highest hexagonal trigonal prismatic TMD materials. The exceptionally large nonlinear response of the TMD Haeckelites can be largely explained by the small band gap.

### TMD Nanotubes

We now turn our attention to the second harmonic response of 1D nanostructures, namely nanotubes. Different types of TMD (10, 0) zigzag nanotubes were calculated. All armchair tubes have no second harmonic response due to the presence of inversion symmetry. The zigzag tubes have direct band gaps at Γ in agreement with previous works^57,58^. The band gaps are 0.20 eV for MoS_2_, 0.42 eV for WS_2_, 0.14 eV for MoSe_2_, 0.27 eV for WSe_2_, 0.44 eV for MoSSe, and 0.63 eV for WSSe. The larger band gaps for the chalcogen alloys agrees with previous results^59^ and is likely due to the increased stability. Zigzag tubes have C_20v_ symmetry with nonzero χ^(2)^ components xyx = zyz, xxy = zzy, yxx = yzz, and yyy^35^.

**Figure 6:**
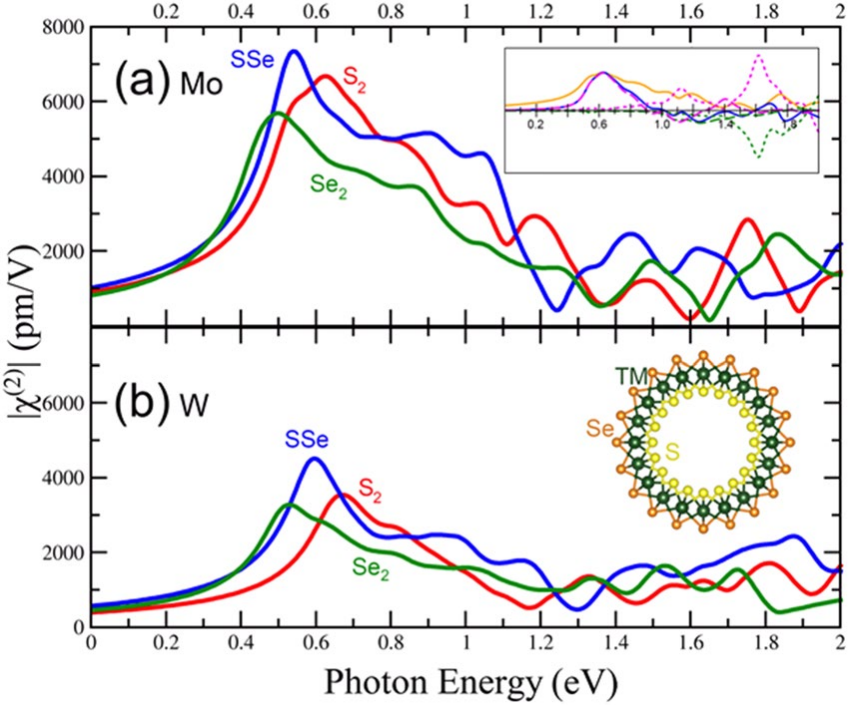
$|{{\rm{X}}}_{xxx}^{(2)}(2\omega ,\omega ,\omega )|$ of (**a**) Mo TMDs tubes and (**b**) W TMDs tubes. Tubes with S2, SSe, and Se2 chalcogens are shown in red, blue, and green respectively. Inset: Comparison of the imaginary components of | χ(2) | (purple) for the MoS2 tube. The total imaginary part is shown in blue. Intraband terms are in green and Interband terms are in red. Dotted lines represent 1ω terms and dashed lines represent 2ω terms.

The calculated χ^(2)^_xxx_(2ω, ω, ω) for the tubes are in Fig. 6a,b for Mo and W respectively. The Mo based tubes all have higher and redshifted response compared to the W based counterparts. Both observations are consistent with the smaller gap of the Mo based tubes. The first peak of the TMSSe tubes clearly falls in between the peaks of the pure phases. The susceptibility is also enhanced with respect to either pure phase, which is in opposition to the general trend of SHG vs band gap found in the flat monolayers. A breakdown of the individual contributions to the |χ^(2)^_xxx_(2ω, ω, ω)| for the MoS_2_ tube is shown in the inset of Fig. 6. The large peak at 0.6 eV is mainly due to the imaginary part, from the 2ω intraband component. The interband components become significant at large photon energies, but the 2ω component is cancelled out by the ω components. The intraband 1ω term is small at all photon energies. Compared to the monolayers, the tubes have a redshifted spectrum with a much higher susceptibility. The susceptibility of the TMD tubes remains high until 1.4–1.7 eV (WS_2_ - MoSSe), at which point the two have comparable responses (See Fig. 6).

### Semiconducting BN and BNC_2_ Monolayers

**Figure 7:**
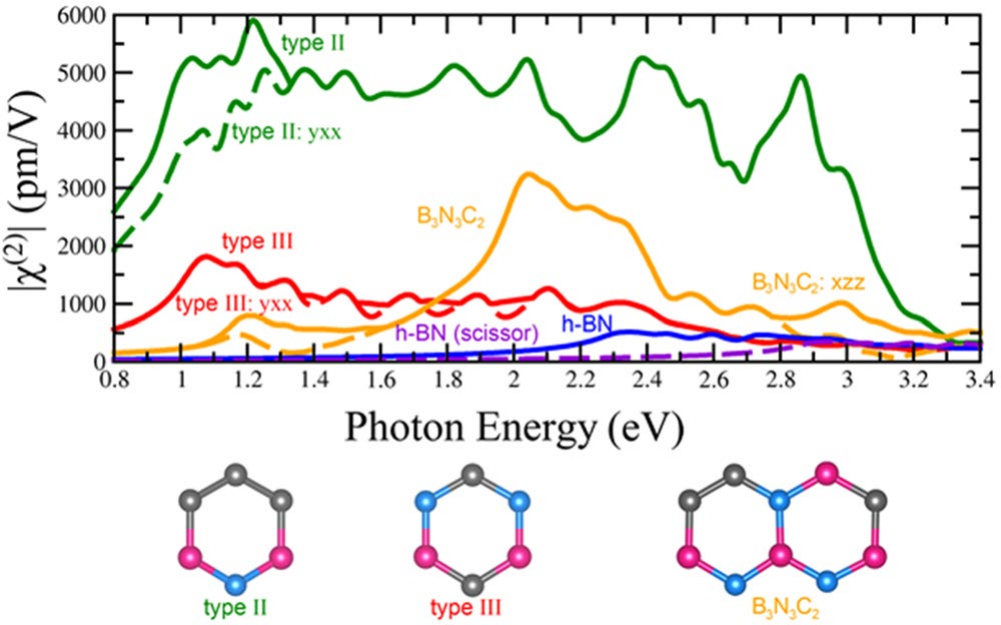
Upper envelopes of |χ^(2)^| for h-BN and BNC alloys: Type II BNC_2_, type III BNC_2_ and B_3_N_3_C_2_ are shown in solid lines. The highest individual component of each alloy is shown with a dashed line. H-BN scissor represents the signal when a shift in the conduction band is carried out to match the experimental band gap. The h-BN signal represents the SHG response for the DFT-LDA band gap. Models are shown where carbon atoms in grey, boron in pink, and nitrogen in blue.

Monolayer BN has space group number 187, P-6m2 with D_3h_ symmetry. The corresponding nonzero χ^(2)^ components are yyy = −yxx = −xxy = −xyx. All BNC have space group number 6, Pm with C_S_ symmetry. The corresponding nonzero χ^(2)^ components are xxx, xyy, xzz, xzx, xxz, yyz, yzy, yxy, yyx, zxx, zyy, zzz, zzx, and zxz^35^. Hexagonal BN (h-BN) has a very small second harmonic response (Fig. 7), especially when compared to the TMDs. The large band gap is one reason why the response is so small. The DFT-LDA band gap for a h-BN monolayer is 4.61 eV while the experimental optical band gap is around 6 eV^24,34^; this is due to the DFT underestimation of the electronic band gap: In order to compensate for this difference, in our calculations for h-BN and BN Schwarzites we have shifted the conduction bands to match the experimental optical band gap. Interestingly, the band gap and the nonlinear optical properties of h-BN can be tuned by alloying BN with Carbon. In this context, we have chosen B_x_N_x_C_y_ alloys with a reduced band gap^60–62^. In reference^60^ three BNC_2_ motifs are considered, type I, type II and type III. Since type I is a metal, we are not going to consider it in our calculations. Type II BNC_2_ motif features alternating zigzag chains of Carbon and Boron Nitride (See Fig. 7) while type III BNC_2_ exhibits alternating stripes of hexagons that contain 2(B-N) and 2(C-C) units and each hexagon has the same amount of Boron and Nitrogen (See Fig. 7). Note that type II and type III have been identified in experimental alloys^63^. Our calculated band gap from the type II is 1.62 eV, in agreement with previously published results^60^ (See supplemental information S5), however our gap for type III is 1.87 eV, much larger than the 0.5 eV reported previously^60^ (See supplemental information S5). A B_3_N_3_C_2_ alloy with a higher band gap of 2.4 eV was also considered (See Fig. 7).

The upper envelope of the calculated second harmonic response χ^(2)^ for the BNC alloys is shown in Fig. 7, all the components are shown in the supplemental information S6. The susceptibility of the pure hexagonal BN monolayer has an onset around half the experimental optical band gap and is very small in magnitude. In Fig. 7 we show both h-BN structures, one without the shift to match the optical gap and another with the scissors operator to match the optical gap to provide a more reliable result. Both BNC_2_ alloys have a giant redshift, as expected from the drastic reduction in the band gap. Moreover, the second harmonic response is greatly enhanced and for the type III and B_3_N_3_C_2_ is comparable to the monolayer TMDs. Surprisingly, the type II SHG susceptibility doubles that of TMDs exhibiting this high response over a wide range of energies from 0.8 to 3.2 eV. Therefore, all these alloys are good candidates for useful nonlinear optical materials. At lower energies, the second order susceptibility of the BC_2_N alloys is significantly higher than that of the monolayer TMDs. At telecom wavelengths, around 1550 nm or 0.8 eV, these alloys have an appreciable second harmonic response. The B_3_N_3_C_2_ case possesses a higher susceptibility than type III at higher photon energies with a larger band gap.

### B_x_N_x_C_y_ Haekelites

**Figure 8:**
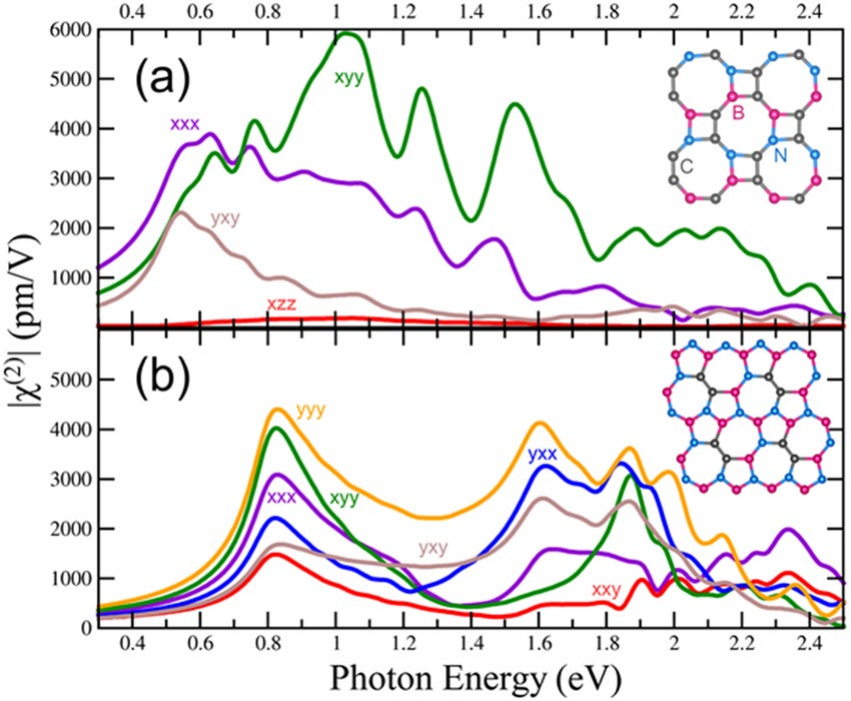
Second order susceptibility for BNC Haeckelites. (**a**) BNC-8–4 Haeckelite (squares and octagons). (**b**) BNC 5–7 Haeckelite (pentagons and heptagons). Carbon atoms in grey, Boron in pink, and Nitrogen in blue.

As seen above in the case of TMDs, Haeckelites have the potential to be extraordinary nonlinear optical structures. The B_x_C_y_N_x_ systems allows us to create 5–7 (pentagons and heptagons) structures in addition to the 8–4 (octagons and squares) motifs mentioned above. The 5–7 BNC Haeckelites has space group number 6, Pm with C_S_ symmetry. The corresponding nonzero χ^(2)^ components are xxx, xyy, xzz, xzx, xxz, yyz, yzy, yxy, yyx, zxx, zyy, zzz, zzx, and zxz^35^. The 8–4 BNC Haekelite has C_2v_ symmetry with space group Pmc2_1_ (number 26). This gives 5 independent nonzero χ^(2)^ components, zzx = zxz, yyx = yxy, xzz, xyy, and xxx^35^. The structures of the 5–7 B_3_C_2_N_3_ and 8–4 B_2_N_2_C_4_ along with their calculated second order suceptibility χ^(2)^ tensor components are shown in Fig. 8a,b respectively. Like the TMD Haeckelites, these materials have exceptionally high second harmonic response. The 5–7 and 8–4 structures have indirect gaps of 1.15 eV and 1.02 eV respectively. The direct gap of the 8–4 Haeckelite is only 2 meV higher than the indirect gap (See supplementary information S5). The smaller gap of the 8–4 is evident in the redshifted χ^(2)^, which has a peak at approximately 0.5 eV, half the band gap. The first peak in the 5–7 Hackelite is not until 0.8 eV, though it starts to show a significant response at around 0.6 eV, half of its band gap.

### BN and BNC_2_ Nanotubes

**Figure 9:**
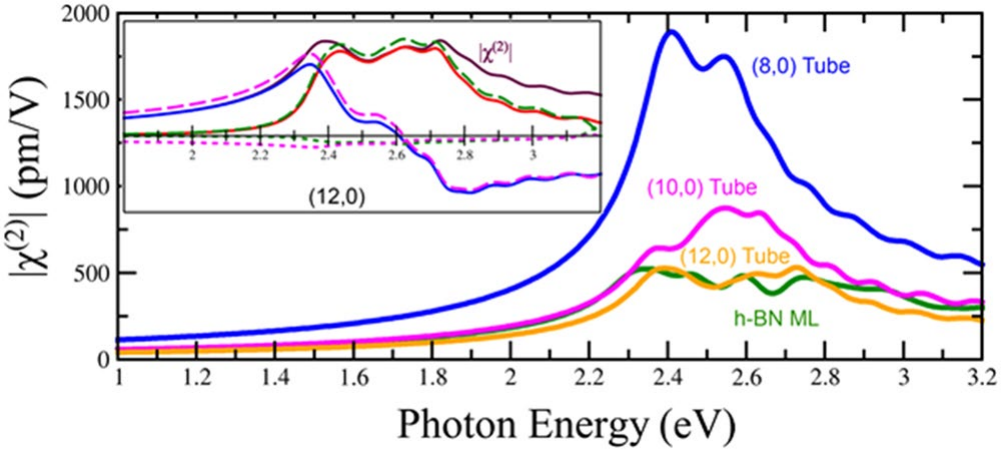
Comparison of | χ^(2)^_yyy_ | for three nanotubes. inset: Comparison of the total real (solid blue) and imaginary parts (solid red) of the (12, 0) BN nanotube. Real (pink) and imaginary (green) interband (dotted) and intraband (dashed) 2ω components are also shown.

The χ^(2)^ of many boron nitride nanotubes have been studied using first principles in such a way that only the direct interband terms were considered^64,65^, as well as through tight binding calculations^66^. All zigzag tubes have C_2nv_ symmetry with nonzero χ^(2)^ components xyx = zyz, xxy = zzy, yxx = yzz, and yyy^35^. As shown in Fig. 9, the SHG susceptibility tends to decrease as the diameter of the tube increases, which is in agreement with the general trend reported by Guo and Lin^64,65^, as well as with our results for TMD nanotubes. Qualitatively, this is a reasonable trend as we would expect the χ^(2)^ susceptibility to approach that of the monolayer as the diameter of the tube approaches infinity. However, a closer look at the interband and intraband terms, shown in Fig. 9 inset, reveals that the intraband terms, which are not considered by Guo and Lin^64,65^, dominate in both the real and imaginary parts of the second order susceptibility χ^(2)^. While the direct LDA band gap of the tubes decreases from 4.3 eV for the (12, 0) tube to 3.7 eV for the (8, 0) tube, the small redshift in the peak positions does not appear to be a result of this change, as the peaks appear at higher energies than half their respective band gaps. Rather, we must attribute most of the behavior to the complex intraband processes that involve movements along bands that interplay with both the linear response and with interband polarization processes^67^.

**Figure 10:**
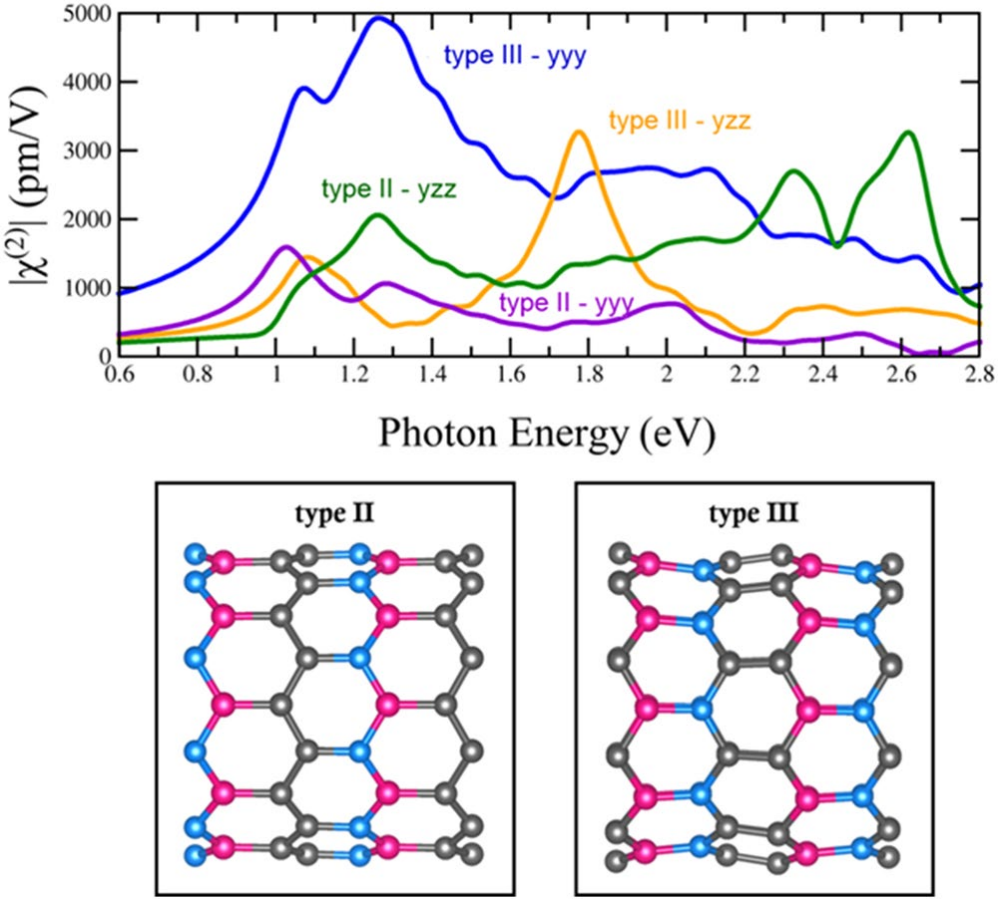
(**a**) Second order susceptibility components for two types of BNC_2_ tubes. (**b**) and (**c**) the BNC_2_ tubes types considered in (**a**): In the type II tube, B-N and C-C bonds are oriented perpendicular to the tube axis, whereas in the type III tube, they are parallel to it. Carbon atoms are grey, Boron atoms are pink, and Nitrogen atoms are blue.

The nonlinear optical properties of two types of (10, 0) BNC_2_ nanotubes were also calculated. The two tubes that were considered, shown in a side view in Fig. 10, have the same stoichiometry but different orientations of C-C and B-N bonds with respect to the tube axis. These two nanotube are derived from the two monolayers considered in Fig. 7, where the type II tube corresponds to a rolled-up type II monolayer and a type III tube corresponds to a type III monolayer. In the type II BNC_2_ tube the C-C and B-N bonds form zigzag chains around the tube whereas in the type III B-N and C-C bonds are parallel to the tube axis. This difference in bond orientation leads to significant differences in the second harmonic response.

Different components of the nonlinear susceptibility χ^(2)^, shown in Fig. 10a, are of interest for the two BNC_2_ nanotubes. For the type II tube, the yzz component has highest susceptibility and for the type III tube, the yyy component is strongest. By far the largest peak is the yyy peak for the type III tube at about 1.25 eV (992 nm). Both the type II and type III tubes have significantly smaller direct LDA band gaps (1.54 eV and 1.53 eV respectively) than the BN nanotubes. The χ^(2)^ susceptibility of the BNC_2_ type III nanotube is both significantly higher and significantly redshifted with respect to that of the BN nanotubes; this redshift can at least partially be attributed to the smaller band gap. The yzz component of the type III tube also has a peak near 1.25 eV, albeit much smaller, but it also has two larger peaks at higher energies that must be attributed to more complex features than the band gap transition.

### Porous BN Structures (Schwarzites)

**Figure 11:**
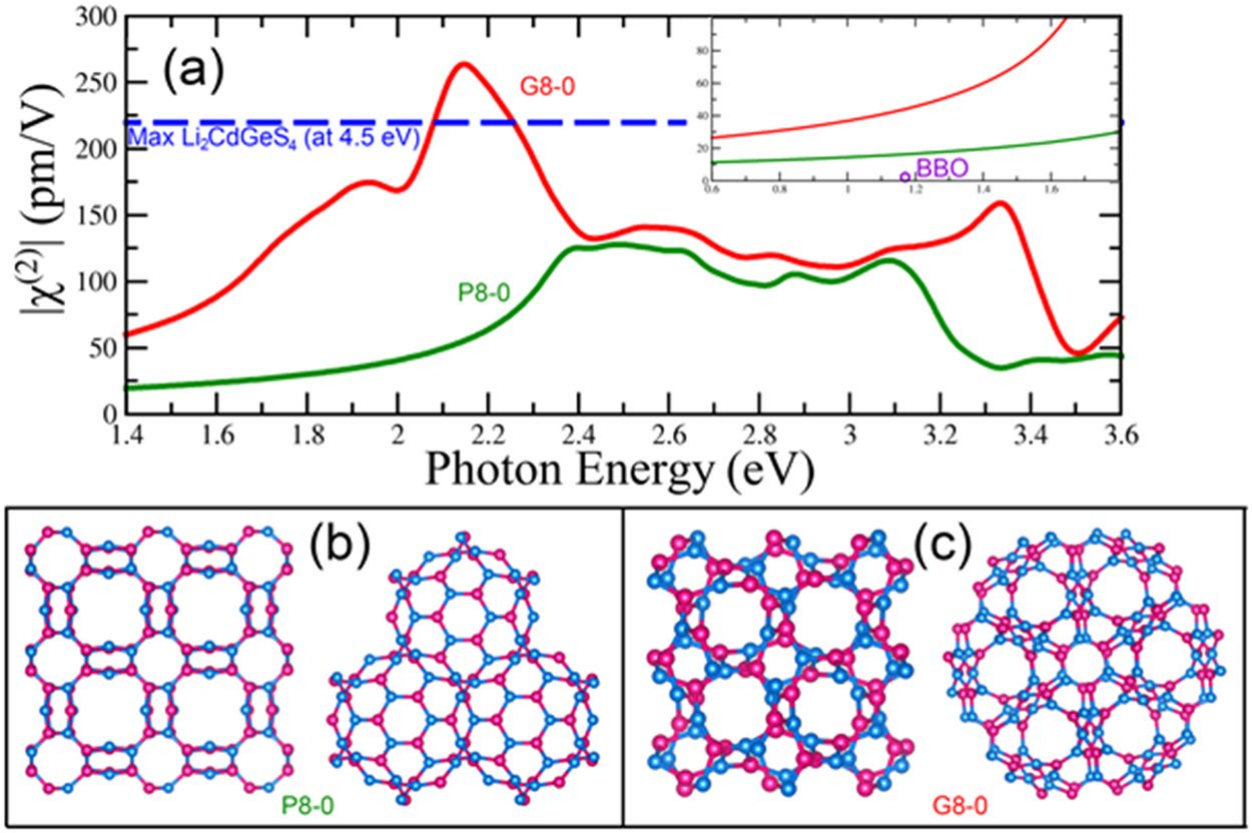
(**a**) Second order susceptibility of the G8–0 and P8-0 Schwarzites. The blue line shows the maximum value of χ^(2)^ of Li_2_CdGeS_4_ one of the highest 3-D materials. The inset shows the signal of BBO. (**b**) and (**c**) Models of the BN Schwarzites: (**b**) P8-0. (**c**) G8-0 (Boron in pink and Nitrogen in blue).

Hypothetical porous 3-D structures with negative Gaussian curvature, named Schwarzites, first proposed by Mackay and Terrones^68^ for carbon materials, have been studied using boron nitride^45^. The negative Gaussain curvature in BN Schwarzites is due to the presence of octagonal rings of alternating Boron and Nitrogen atoms (see Fig. 11). Following the notation used in reference^69^, the G8-0 and P8-0 structures exhibit LDA direct band gaps at Γ of 2.72 eV and 3.16 eV respectively (See supplemental information S7) which have been shifted equivalently to consider the experimental optical band gap of h-BN. These structures possess T_d_ symmetry which gives one independent χ^(2)^ term, xyz. The P structure has space group number 217, I-43m, while the G structure has space group number 199, I2_1_3. Therefore, the P structure has T_d_ symmetry, which has nonzero χ^(2)^ components xyz = xzy = yzx = yxz = zxy = zyx^35^. The G structure exhibits T symmetry which has nonzero χ^(2)^ components xyz = yzx = zxy and xzy = yxz = zyx^35^. For the special case of SHG, these two sets of χ^(2)^ components for the G structure are equal. The calculated χ^(2)^_xyz_ for the porous structures is shown in Fig. 11. Along with a redshift, due to the decreased band gap of the porous structures, an enhanced SHG susceptibility is observed. The signal of the G8-0 is higher than the maximum susceptibility reported for Li_2_CdGeS_4_, one of the highest large band gap χ^(2)^ 3D-materials found so far^46^ (See Fig. 11). The P8-0 structure, shows a lower intensity than the G8-0, but with a nice plateau between 2 and 2.6 eV which could be useful for nonlinear optics applications. Another scissor shift was applied to the G8-0 to increase the gap to be the same as the P8-0. The resulting χ^(2)^ is diminished, but still larger than that of P8-0 demonstrating that the difference in atomic structure, not just electronic structure, is responsible for the improved nonlinear optical properties.

Note that by introducing negative Gaussian curvature via BN octagonal rings, 3-D porous materials with worthy nonlinear optical properties can be generated. As in Carbon, different types and sizes of BN Schwarzites can be calculated, the challenge is thus their synthesis. The 3-D nature of this structure allows for a comparison to conventional NLO crystals, since χ^(2)^ is only well defined in 3-D materials. BBO is a common NLO crystal with a χ^(2)^ of 2.7 pm/V at 1.17 eV, which is an order of magnitude lower than that of these BN Schwarzites.

## Conclusion

In conclusion, we have shown that by alloying and introducing curvature in 2-D layered materials new robust nonlinear optical systems can be obtained. Chalcogen alloys possess high χ^(2)^ intensities, being the highest the tungsten based alloy WSSe(V) at around 2 eV, however pure MoSTe(H) reveals the highees χ^(2)^ at low photon energy levels in the range 0.8eV–1.6 eV. Transition metal alloys of the type MoWS or MoWSe reveal lower χ^(2)^ than chalcogen alloys, therefore, chalcogen based alloys are better candidates for new nonlinear optical devices. Regarding TMD nanotube alloys, we have demonstrated that Mo based nanotubes possess higher χ^(2)^ than W based: Both systems show lower band gaps than the flat monolayers and higher χ^(2)^ at lower photon energies. TMD alloy nanotubes look promising for nonlinear optical devices at low energies. Though MoS_2_ and WS_2_ nanotubes were synthesized 25 years ago^70–72^, new efforts in the synthesis need to be made, and new integration techniques need to be implemented to use their nonlinear optical properties in new devices. The same applies to BN zigzag and BNC_2_ nanotubes which exhibit high χ^(2)^, though in BNC_2_ nanotubes the χ^(2)^ is higher than in pure BN zigzag tubes. Our results indicate that flat layers of BNC alloys possess much higher χ^(2)^ intensities than pure flat h-BN. To synthesize BNC alloys we suggest the strategy to start from already grown chemical vapor deposition BN monolayers and then add the carbon from a carbon source. Starting from graphene has leaded to segregation of BN and Carbon^73^ which may not be suitable for nonlinear optical properties. Surprisingly, TMD Haeckelites exhibit the highest χ^(2)^ of all the systems we have calculated. In this context, among the BN systems studied, BN Haeckelites also reveal a very high χ^(2)^, not as high as in TMD. We have found that the introduction of defective patches in an ordered way enhances the nonlinear optical response. In fact, the defects in the Haeckelites can be seen as a combination of positive and negative Gaussian curvature patches in the same proportion to balance the curvatures producing a flat layer^41,42^. Experimentally, ion irradiation of TMD and BN alloy systems, at high temperatures, might lead to structures with Haeckelite patches that could greatly increase the χ^(2)^. It is worth noticing that the 8–4 patches and 5–7 patches have been observed in grain boundaries in MoS_2_^74,75^. Our results disclose that negative Gaussian curvature BN Schwarzites (Porous BN) exhibit higher χ^(2)^ than 3-D known systems. In general, we have shown that higher χ^(2)^ responses correspond to lower band gap systems, a similar trend is found in 3-D semiconductors^76–78^ and non-alloyed TMDs^38,40^, but also intraband and interband effects are relevant. In addition, our results reveal that curvature plays a an important role in the χ^(2)^ response.

## Methods

Density Functional calculations are preformed using the local density appoximation (LDA)^39^ in the ABINIT code^79,80^ with the projector augmented wave (PAW) potential method^81–83^. The PAW potentials for the transition metals Mo and Nb include (4s, 4p, 5s, 4d) electrons in the valence, while the potentials for W and Ta include (5s, 5p, 6s, 5d) electrons in the valence. For the chalcogens S and Se, the PAW potentials include (3s, 3p) and (4s, 4p) electrons in the valence respectively. For the first-row elements B, C, and N, the PAW potentials include (2s, 2p) electrons in the valence. The wave functions are expanded in a plane wave basis up to a cutoff energy of 408 eV. The theoretically determined lattice constants were used for all materials. A Γ centered 12 × 12 × 1 k-point grid is used for the ground state calculations for the monolayer unit cells of the simple hexagonal lattice, which are 20 Å and 15 Å long in the perpendicular direction for the transition metal and B_x_N_y_C_z_ layered materials respectively. Isolated nanotubes were placed in square lattices with more than 15 Å separating the tubes. Seven k-points were used in the periodic tube direction for the calculation of the ground state. The porous P8-0 and G8-0 BN Schwarzites are evaluated with a 6 × 6 × 6 and 5 × 5 × 5 k-point mesh, respectively. All atomic structures are relaxed until the forces are less than 0.01 eV/Å. For h-BN and the BN Schwarzites, a shift of the conduction band is carried out to match the experimental optical band gap of h-BN. In the case of TMDs this shift is not necessary since LDA provides a good approximation of the optical band gap.

We calculate the second order susceptibility χ^(2)^(2ω, ω, ω) within the independent particle approximation. The expression for χ^(2)^(2ω, ω, ω) is derived within the dipole approximation^37^ following the work of Ghahramani *et al*.^84–87^ as implemented in the ABINIT code. The resulting three terms represent the interband transitions, the intraband transitions, and the modulation of the interband by the intraband transitions:

1 $$\begin{array}{rcl}{\chi }_{{inter}}^{abc}(2\omega ,\omega ,\omega ) & = & \frac{1}{\Omega }\sum _{nmlk}{W}_{{\boldsymbol{k}}}\{\frac{2{r}_{nm}^{a}\{{r}_{ml}^{b}{r}_{ln}^{c}\}}{({\omega }_{\mathrm{ln}}-{\omega }_{ml})({\omega }_{{mn}}-2\omega )}-\frac{1}{({\omega }_{{mn}}-\omega )}\\  &  & [\frac{{r}_{lm}^{c}\{{r}_{mn}^{a}{r}_{nl}^{b}\}}{({\omega }_{{nl}}-{\omega }_{mn})}-\frac{{r}_{nl}^{b}\{{r}_{lm}^{c}{r}_{mn}^{a}\}}{({\omega }_{{lm}}-{\omega }_{mn})}]\}\end{array}$$

2 $$\begin{array}{rcl}{\chi }_{{intra}}^{abc}(2\omega ,\omega ,\omega ) & = & \frac{1}{\Omega }\sum _{{\boldsymbol{k}}}{W}_{{\boldsymbol{k}}}\{\sum _{nml}\frac{[{\omega }_{ln}{r}_{nl}^{b}\{{r}_{lm}^{c}{r}_{mn}^{a}\}-{\omega }_{ml}{r}_{lm}^{c}\{{r}_{mn}^{a}{r}_{nl}^{b}\}]}{{\omega }_{mn}^{2}({\omega }_{{mn}}-\omega )}-8i\sum _{nm}\frac{{r}_{nm}^{a}\{{{\rm{\Delta }}}_{mn}^{b}{r}_{mn}^{c}\}}{{\omega }_{mn}^{2}-2\omega }\\  &  & +2\sum _{nml}\frac{{r}_{nm}^{a}\{{r}_{ml}^{b}{r}_{\mathrm{ln}}^{c}\}({\omega }_{{ml}}-{\omega }_{\mathrm{ln}})}{{\omega }_{mn}^{2}({\omega }_{{mn}}-2\omega )}\}\end{array}$$

3 $$\begin{array}{rcl}{\chi }_{{mod}}^{abc}(2\omega ,\omega ,\omega ) & = & \frac{1}{2\Omega }\sum _{{\boldsymbol{k}}}{W}_{{\boldsymbol{k}}}\{\sum _{nml}\frac{[{\omega }_{nl}{r}_{lm}^{a}\{{r}_{mn}^{b}{r}_{nl}^{c}\}-{\omega }_{lm}{r}_{nl}^{a}\{{r}_{lm}^{b}{r}_{mn}^{c}\}]}{{\omega }_{mn}^{2}({\omega }_{{mn}}-\omega )}\\  &  & -\,i\sum _{nm}\frac{{r}_{nm}^{a}\{{r}_{mn}^{b}{{\rm{\Delta }}}_{mn}^{c}\}}{{\omega }_{mn}^{2}({\omega }_{mn}-2\omega )}\}\end{array}$$

where a, b, and c are Cartesian components and W_k_ is the weight of the k point, n are valence states, m are conduction states, and l are all states (l ≠ m, n). r_nm_ are the position matrix elements given by ${r}_{nm}=\frac{{v}_{nm}}{i{\omega }_{nm}}$ where ${v}_{nm}=\langle {\psi }_{m}|-i\nabla |{\psi }_{n}\rangle$ and ω_mn_ = ω_m_ − ω_n_ with the energy of band n being *ℏ*ω_n_. With ${{\rm{\Delta }}}_{nm}={v}_{nm}-{v}_{mn}$ and the curly brackets indicates a symmetrization with respect to Cartesian components $\{{r}_{ml}^{b}{r}_{\mathrm{ln}}^{c}\}=\frac{1}{2}({r}_{{ml}}^{b}{r}_{\mathrm{ln}}^{c}+{r}_{{ml}}^{c}{r}_{\mathrm{ln}}^{b})$. 65 and 100 conduction bands are included for the monolayers and nanomaterials exhibiting curvature respectively. A k-point mesh of 48 × 48 × 1 and 48 × 1 × 1 is used to obtain the wave functions for the optical calculation for the monolayer unit cells and nanotubes respectively. A 6 × 6 × 6 and 5 × 5 × 5 k-point mesh is used for the porous P8-0 and G8-0 BN Schwarzites respectively. A smearing of 0.0544 eV is applied to the optical spectrum to obtain smooth plots. The y-direction is chosen to be along the armchair direction for all hexagonal systems.

Surface susceptibilities can be obtained by considering the 2D material to by infinitesimally thin. Therefore, we would have to multiply by the size of the simulation cell in the perpendicular direction. A factor of 2,000 and 1.500 is needed to obtain the spectrum in the units of pm^2^/V for the TMDs and BN, respectively. We choose to report effective bulk susceptibility by assuming an effective layer thickness. In this case, we multiply our calculated values by a dimensionless quantity that represents the fraction of our cell that contains active material to retain the unit of pm/V. This dimensionless quantity is l_z_/d_eff_, where d_eff_ is the effective layer thickness and l_z_ is the length of the cell in the z direction. Using the experimental lattice constants as a reference, 6.3 Å and 3.3 Å are chosen for the monolayer thickness for the TMDs and BN structures respectively. To obtain the surface SHG (units pm^2^/V), multiply the y-axis by 630 and 330 for the TMD and BN materials, respectively. Extending this concept to the one dimensional nanotubes, we need an effective area. The dimensionless quantity for the tubes is A_cell_/A_tube_, where A_tube_ is the cross-sectional area of the tube defined by the circle of the outermost atoms and A_cell_ is the area of unit the cell perpendicular to the tube axis. The diameters of the TMD nanotubes very only slightly, so we use a constant value of 17.5 Å for the diameter all TMD nanotubes.

## Electronic supplementary material

Supplementary Information

## References

[1] Wang QA, Kalantar-Zadeh K, Kis A, Coleman JN, Strano MS (2012). Electronics and optoelectronics of two-dimensional transition metal dichalcogenides. Nat. Nanotechnol..

[2] Xu M, Liang T, Shi M, Chen H (2013). Graphene-Like Two-Dimensional Materials. Chem. Rev..

[3] Huang X, Zeng Z, Zhang H (2013). Metal dichalcogenide nanosheets: preparation, properties and applications. Chem. Soc. Rev..

[4] Balendhran S (2013). Two-Dimensional Molybdenum Trioxide and Dichalcogenides. Adv. Funct. Mater..

[5] Mak, K. F., Lee, C., Hone, J., Shan, J. & Heinz, T. F. Atomically Thin MoS_2_: A New Direct-Gap Semiconductor. *Phys. Rev. Lett*. **105**, 10.1103/PhysRevLett.105.136805 (2010). 10.1103/PhysRevLett.105.136805 21230799

[6] Zhong L (2016). Defect engineering of two-dimensional transition metal dichalcogenides. 2D Materials.

[7] Cao T (2012). Valley-selective circular dichroism of monolayer molybdenum disulphide. Nat Commun.

[8] Kim J (2014). Ultrafast generation of pseudo-magnetic field for valley excitons in WSe2 monolayers. Science.

[9] Mak KF, He KL, Shan J, Heinz TF (2012). Control of valley polarization in monolayer MoS_2_ by optical helicity. Nat. Nanotechnol..

[10] Mak KF, He KL, Shan J, Heinz TF (2012). Control of valley polarization in monolayer MoS2 by optical helicity. Nat Nano.

[11] Xiao, D., Liu, G. B., Feng, W. X., Xu, X. D. & Yao, W. Coupled Spin and Valley Physics in Monolayers of MoS_2_ and Other Group-VI Dichalcogenides. *Phys. Rev. Lett*. **108**, 196802 10.1103/PhysRevLett.108.196802 (2012). 10.1103/PhysRevLett.108.196802 23003071

[12] Zeng HL, Dai JF, Yao W, Xiao D, Cui XD (2012). Valley polarization in MoS_2_ monolayers by optical pumping. Nat. Nanotechnol..

[13] Manca, M. *et al*. Enabling valley selective exciton scattering in monolayer WSe2 through upconversion. *Nature Communications* **8**, 10.1038/ncomms14927 (2017). 10.1038/ncomms14927 PMC5382264 28367962

[14] Singh, A. *et al*. Long-Lived Valley Polarization of Intravalley Trions in Monolayer WSe2. *Phys. Rev. Lett*. **117**, 10.1103/PhysRevLett.117.257402 (2016). 10.1103/PhysRevLett.117.257402 28036191

[15] Ramasubramaniam, A. Large excitonic effects in monolayers of molybdenum and tungsten dichalcogenides. *Phys. Rev. B* **86**, 10.1103/PhysRevB.86.115409 (2012).

[16] Ye ZL (2014). Probing excitonic dark states in single-layer tungsten disulphide. Nature.

[17] Yu YF (2015). Equally Efficient Inter layer Exciton Relaxation and Improved Absorption in Epitaxial and Nonepitaxial MoS2/WS2 Heterostructures. Nano Lett..

[18] Palummo M, Bernardi M, Grossman JC (2015). Exciton Radiative Lifetimes in Two-Dimensional Transition Metal Dichalcogenides. Nano Lett..

[19] Hill HM (2015). Observation of Excitonic Rydberg States in Monolayer MoS2 and WS2 by Photoluminescence Excitation Spectroscopy. Nano Lett..

[20] Kim MS (2016). Biexciton Emission from Edges and Grain Boundaries of Triangular WS2 Monolayers. Acs Nano.

[21] Jones AM (2016). Excitonic luminescence upconversion in a two-dimensional semiconductor. Nature Physics.

[22] Kozawa D (2016). Evidence for Fast Interlayer Energy Transfer in MoSe2/WS2 Heterostructures. Nano Lett..

[23] Molas, M. R. *et al*. Brightening of dark excitons in monolayers of semiconducting transition metal dichalcogenides. *2d Materials* **4**, 10.1088/2053-1583/aa5521 (2017).

[24] Li Y (2013). Probing Symmetry Properties of Few-Layer MoS2 and h-BN by Optical Second-Harmonic Generation. Nano Letters.

[25] Zeng, H. L. *et al*. Optical signature of symmetry variations and spin-valley coupling in atomically thin tungsten dichalcogenides. *Sci. Rep*. **3**, 10.1038/srep01608 (2013). 10.1038/srep01608 PMC3622914 23575911

[26] Kumar, N. *et al*. Second harmonic microscopy of monolayer MoS2. *Phys. Rev. B* **87**, 10.1103/PhysRevB.87.161403 (2013).

[27] Malard, L. M., Alencar, T. V., Barboza, A. P. M., Mak, K. F. & de Paula, A. M. Observation of intense second harmonic generation from MoS2 atomic crystals. *Phys. Rev. B* **87**, 10.1103/PhysRevB.87.201401 (2013).

[28] Wang, G. *et al*. Giant Enhancement of the Optical Second-Harmonic Emission of WSe2 Monolayers by Laser Excitation at Exciton Resonances. *Phys. Rev. Lett*. **114**, 10.1103/PhysRevLett.114.097403 (2015). 10.1103/PhysRevLett.114.097403 25793850

[29] Hsu WT (2014). Second Harmonic Generation from Artificially Stacked Transition Metal Dichalcogenide Twisted Bilayers. Acs Nano.

[30] Janisch, C. *et al*. Extraordinary Second Harmonic Generation in Tungsten Disulfide Monolayers. *Sci. Rep*. **4**, 10.1038/srep05530 (2014). 10.1038/srep05530 PMC4078302 24984953

[31] Yi F (2016). Optomechanical Enhancement of Doubly Resonant 2D Optical Nonlinearity. Nano Lett..

[32] Day JK, Chung MH, Lee YH, Menon VM (2016). Microcavity enhanced second harmonic generation in 2D MoS2. Optical Materials Express.

[33] Le CT (2017). Impact of Selenium Doping on Resonant Second-Harmonic Generation in Monolayer MoS2. Acs Photonics.

[34] Kim C-J (2013). Stacking Order Dependent Second Harmonic Generation and Topological Defects in h-BN Bilayers. Nano Letters.

[35] Boyd, R. W. Nonlinear Optics. 3 edn, (Academic Press, 2008).

[36] Luppi, E., Hubener, H. & Veniard, V. Ab initio second-order nonlinear optics in solids: Second-harmonic generation spectroscopy from time-dependent density-functional theory. *Phys. Rev. B* **82**, 10.1103/PhysRevB.82.235201 (2010).

[37] Sharma S, Ambrosch-Draxl C (2004). Second-Harmonic Optical Response from First Principles. Phys. Scr..

[38] Grüning M, Attaccalite C (2014). Second harmonic generation in h-BN and MoS_2_ monolayers: Role of electron-hole interaction. Phys. Rev. B.

[39] Perdew JP, Wang Y (1992). Accurate and simple analytic representation of the electron-gas correlation energy. Phys. Rev. B.

[40] Trolle ML, Seifert G, Pedersen TG (2014). Theory of excitonic second-harmonic generation in monolayer MoS_2_. Phys. Rev. B.

[41] Crespi VH, Benedict LX, Cohen ML, Louie SG (1996). Prediction of a pure-carbon planar covalent metal. Phys. Rev. B.

[42] Terrones H (2000). New Metallic Allotropes of Planar and Tubular Carbon. Phys. Rev. Lett..

[43] Terrones H, Terrones M (2014). Electronic and vibrational properties of defective transition metal dichalcogenide Haeckelites: new 2D semi-metallic systems. 2D Materials.

[44] Lenosky T, Gonze X, Teter M, Elser V (1992). Energetics of negatively curved graphitic carbon. Nature.

[45] Gao P (2017). BN-schwarzite: novel boron nitride spongy crystals. Phys. Chem. Chem. Phys..

[46] Reshak AH (2016). Revealing the origin of the strong second harmonic generation of Li2CdXS4 and Li2CdXS4 (X = Ge or Sn). J. Appl. Phys..

[47] Lin K-H, Weng S-W, Lyu P-W, Tsai T-R, Su W-B (2014). Observation of optical second harmonic generation from suspended single-layer and bi-layer graphene. Appl. Phys. Lett..

[48] Zhang J (2017). Janus Monolayer Transition-Metal Dichalcogenides. ACS Nano.

[49] Azizi A (2016). Spontaneous Formation of Atomically Thin Stripes in Transition Metal Dichalcogenide Monolayers. Nano Lett..

[50] Chen Y (2013). Tunable Band Gap Photoluminescence from Atomically Thin Transition-Metal Dichalcogenide Alloys. ACS Nano.

[51] Gan, L.-Y., Zhang, Q., Zhao, Y.-J., Cheng, Y. & Schwingenschlögl, U. Order-disorder phase transitions in the two-dimensional semiconducting transition metal dichalcogenide alloys Mo1−xWxX2 (X = S, Se, and Te). **4**, 6691, 10.1038/srep06691 (2014). 10.1038/srep06691 PMC4204064 25331363

[52] Kutana A, Penev ES, Yakobson BI (2014). Engineering electronic properties of layered transition-metal dichalcogenide compounds through alloying. Nanoscale.

[53] Wang G (2015). Exciton states in monolayer MoSe 2: impact on interband transitions. 2D Materials.

[54] Kim DH, Lim D (2015). Optical second-harmonic generation in few-layer MoSe2. Journal of the Korean Physical Society.

[55] Le CT (2016). Nonlinear optical characteristics of monolayer MoSe_2_. Annalen der Physik.

[56] Wang C-Y, Guo G-Y (2015). Nonlinear Optical Properties of Transition-Metal Dichalcogenide MX_2_ (M = Mo, W; X = S, Se) Monolayers and Trilayers from First-Principles Calculations. The Journal of Physical Chemistry C.

[57] Seifert G, Terrones H, Terrones M, Jungnickel G, Frauenheim T (2000). Structure and Electronic Properties of MoS_2_ Nanotubes. Phys. Rev. Lett..

[58] Seifert G, Terrones H, Terrones M, Jungnickel G, Frauenheim T (2000). On the electronic structure of WS_2_ nanotubes. Solid State Commun..

[59] Zhao W, Li Y, Duan W, Ding F (2015). Ultra-stable small diameter hybrid transition metal dichalcogenide nanotubes X-M-Y (X, Y = S, Se, Te; M = Mo, W, Nb, Ta): a computational study. Nanoscale.

[60] Liu AY, Wentzcovitch RM, Cohen ML (1989). Atomic arrangement and electronic structure of BC_2_N. Phys. Rev. B.

[61] Peng Q, De S (2012). Tunable band gaps of mono-layer hexagonal BNC heterostructures. Physica E: Low-dimensional Systems and Nanostructures.

[62] Shi Z, Kutana A, Yakobson BI (2015). How Much N-Doping Can Graphene Sustain?. The Journal of Physical Chemistry Letters.

[63] Lu, J. *et al*. Order–disorder transition in a two-dimensional boron–carbon–nitride alloy. **4**, 2681, 10.1038/ncomms3681https://www.nature.com/articles/ncomms3681#supplementary-information (2013). 10.1038/ncomms3681 24157959

[64] Guo GY, Lin JC (2005). Second-harmonic generation and linear electro-optical coefficients of BN nanotubes. Phys. Rev. B.

[65] Guo GY, Lin JC (2008). Erratum: Second-harmonic generation and linear electro-optical coefficients of BN nanotubes [Phys. Rev. B 72, 075416 (2005)]. Phys. Rev..

[66] Margulis VA, Muryumin EE, Gaiduk EA (2010). Second-order nonlinear optical response of zigzag BN single-walled nanotubes. Phys. Rev. B.

[67] Rashkeev SN, Limpijumnong S, Lambrecht WRL (1999). Second-harmonic generation and birefringence of some ternary pnictide semiconductors. Phys. Rev. B.

[68] Mackay AL, Terrones H (1991). Diamond from graphite. Nature.

[69] Miller DC, Terrones M, Terrones H (2016). Mechanical properties of hypothetical graphene foams: Giant Schwarzites. Carbon.

[70] Tenne R, Margulis L, Genut M, Hodes G (1992). Polyhedral and Cylindrical Structures of Tungsten Disulfide. Nature.

[71] Tenne R, Margulis L, Hodes G (1993). Fullerene-Like Nanocrystals of Tungsten Disulfide. Adv. Mater..

[72] Rapoport L, Fleischer N, Tenne R (2005). Applications of WS_2_ (MoS_2_) inorganic nanotubes and fullerene-like nanoparticles for solid lubrication and for structural nanocomposites. J. Mater. Chem..

[73] Ci, L. *et al*. Atomic layers of hybridized boron nitride and graphene domains. *Nat Mater* **9**, 430-435, http://www.nature.com/nmat/journal/v9/n5/abs/nmat2711.html#supplementary-information (2010). 10.1038/nmat2711 20190771

[74] Najmaei, S. *et al*. Vapour phase growth and grain boundary structure of molybdenum disulphide atomic layers. *Nat Mater* **12**, 754–759, 10.1038/nmat3673http://www.nature.com/nmat/journal/v12/n8/abs/nmat3673.html#supplementary-information (2013). 10.1038/nmat3673 23749265

[75] van der Zande AM (2013). Grains and grain boundaries in highly crystalline monolayer molybdenum disulphide. Nature Materials.

[76] Chang EK, Shirley EL, Levine ZH (2001). Excitonic effects on optical second-harmonic polarizabilities of semiconductors. Phys. Rev. B.

[77] Kang L (2015). Metal Thiophosphates with Good Mid-infrared Nonlinear Optical Performances: A First-Principles Prediction and Analysis. JACS.

[78] Rashkeev SN, Lambrecht WRL (2001). Second-harmonic generation of I-III-VI_2_ chalcopyrite semiconductors: Effects of chemical substitutions. Phys. Rev. B.

[79] Gonze X (2016). Recent developments in the ABINIT software package. Comput. Phys. Commun..

[80] Torrent M, Jollet F, Bottin F, Zérah G, Gonze X (2008). Implementation of the projector augmented-wave method in the ABINIT code: Application to the study of iron under pressure. Computational Materials Science.

[81] Kresse G, Joubert D (1999). From ultrasoft pseudopotentials to the projector augmented-wave method. Phys. Rev. B.

[82] Blöchl PE (1994). Projector augmented-wave method. Phys. Rev. B.

[83] Jollet F, Torrent M, Holzwarth N (2014). Generation of Projector Augmented-Wave atomic data: A 71 element validated table in the XML format. Comput. Phys. Commun..

[84] Ghahramani E, Moss DJ, Sipe JE (1990). Linear optical properties of strained (Si_n_) /(Ge)_n_ superlattices on (001) Si substrates. Phys. Rev. B.

[85] Ghahramani E, Moss DJ, Sipe JE (1991). Full-band-structure calculation of second-harmonic generation in odd-period strained (Si)_n_/(Ge)_n_ superlattices. Phys. Rev. B.

[86] Ghahramani E, Moss DJ, Sipe JE (1991). Linear and nonlinear optical properties of (GaAs)_m_/(AlAs)_n_ superlattices. Phys. Rev. B.

[87] Ghahramani E, Sipe JE (1992). Full-band-structure calculation of substrates E- >2(w) and X->(2)(−2w; w, w) for (Ga)n/(GaP)n(n = 1, 2) superlattices on GaS(001) substrates. Phys. Rev. B.

