# Supplementary material for: Large second harmonic generation in alloyed TMDs and boron nitride nanostructures
Source: Sci Rep. 2018 Jul 4;8:10118. doi: 10.1038/s41598-018-27702-9 (PMC6031671; doi:10.1038/s41598-018-27702-9)
Supplement: Supplementary file 1 — Supplementary Information [file 41598_2018_27702_MOESM1_ESM.docx]

**Supplementary Information for the manuscript Large second harmonic generation in alloyed TMDs and boron nitride nanostructures**

Michael C. Lucking, Kory Beach, and Humberto Terrones*

*Department of Physics, Applied Physics, and Astronomy, Rensselaer Polytechnic Institute, Troy, New York 12180, United States*


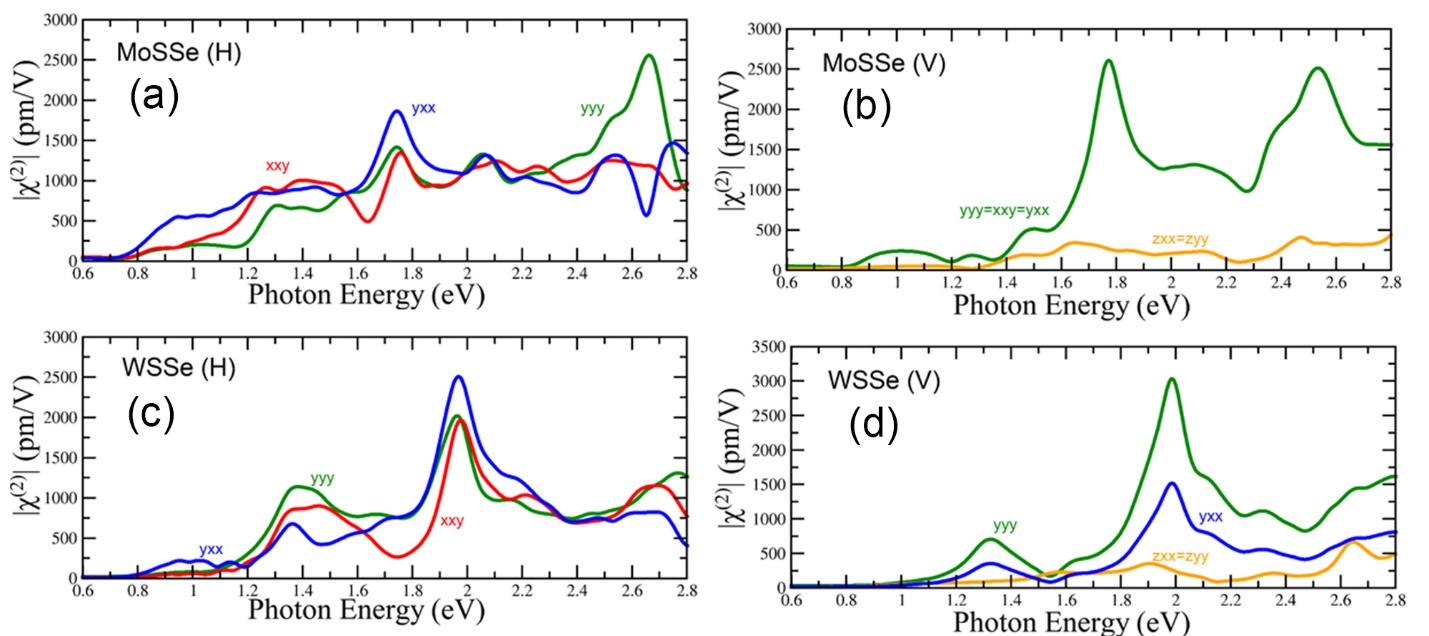


Figure S1. |χ^(2)^| components of Mo and W chalcogen alloys. (a) MoSSe(H); (b) MoSSe(V);

(c) WSSe(H); (d) WSSe(V).


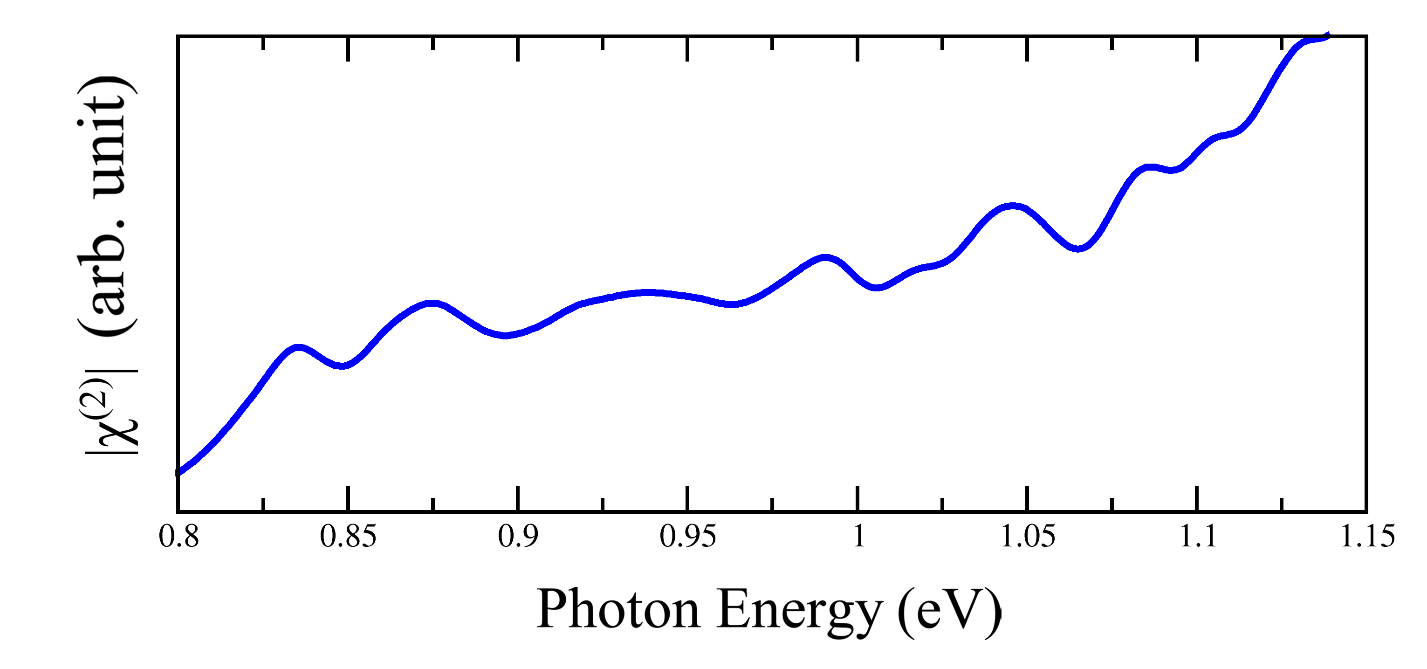


Figure S2. Low energy region of MoSe_2_ SHG spectrum with lower (0.0136 eV) smearing. Peaks at 0.83, 0.93, and 1 eV agree with the experimental spectrum. However, experiment sees the spectrum decay more at 0.87eV and past 1.03 eV.


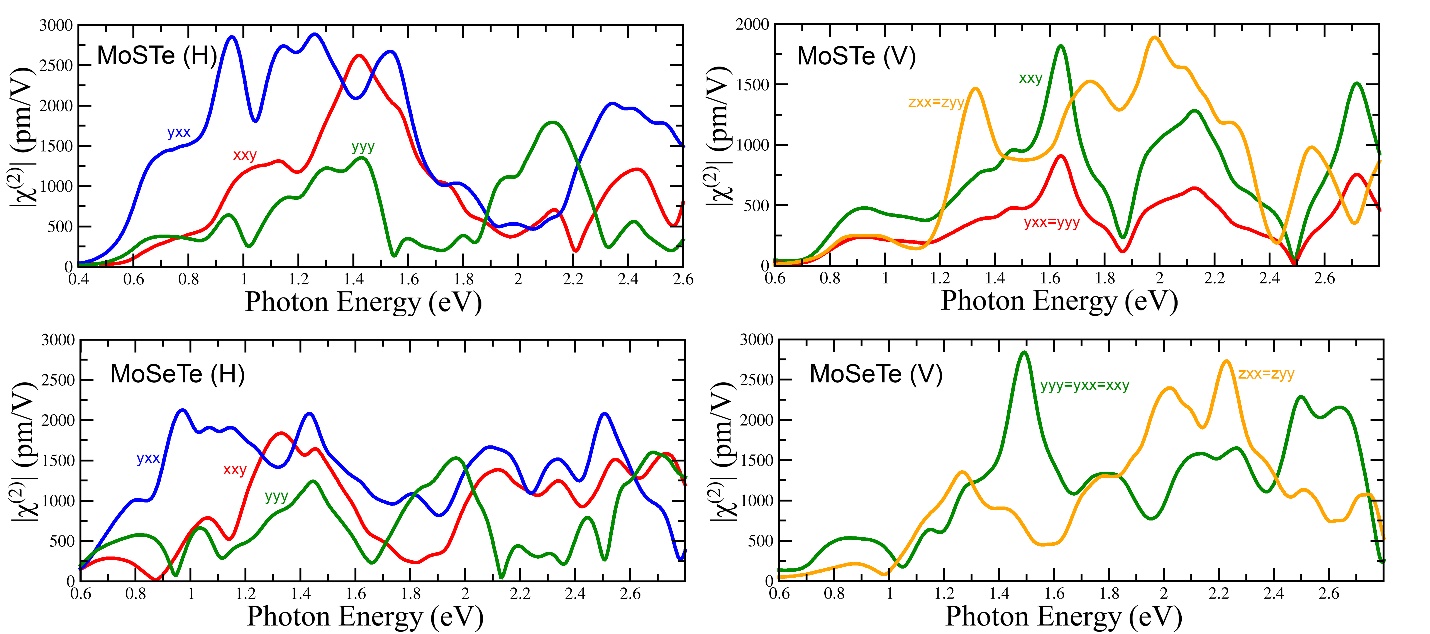


Figure S3. |χ^(2)^| components for MoSTe(H), MoSTe(V), MoSeTe(H) and MoSeTe(V).


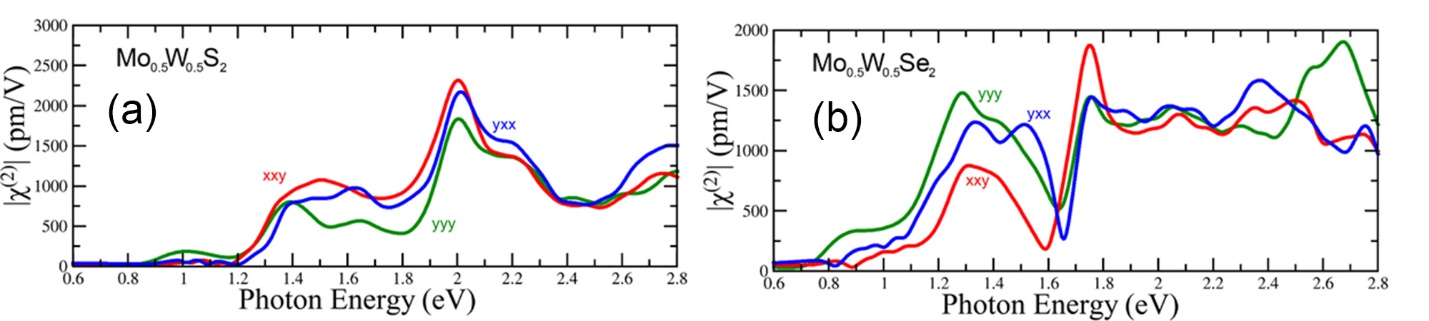


Figure S4. |χ^(2)^| components of the SHG tensor for the transition metal alloys: (a) Mo_0.5_W_0.5_ S_2_ ;

(b) Mo_0.5_W_0.5_ Se_2_ alloy.


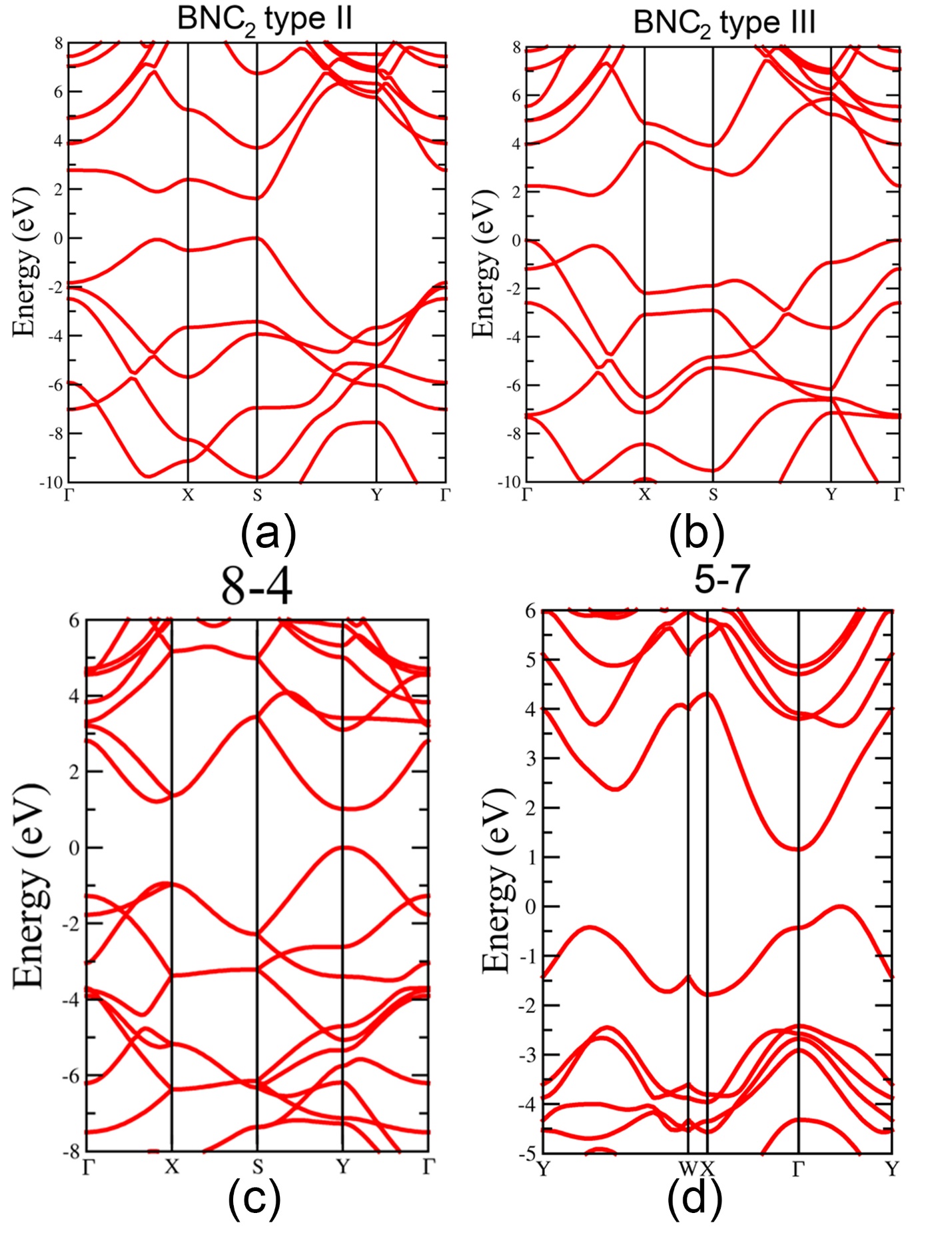


Figure S5. Band structures of BNC alloys: (a) BNC_2_ alloy (Type 2); (b) BNC_2_ alloy (Type III); (c) Haeckelite B_2_N_2_C_4_ 8-4; (d) Haeckelite B_3_N_3_C_2_ 5-7. Valence band maximum is set to 0 eV.


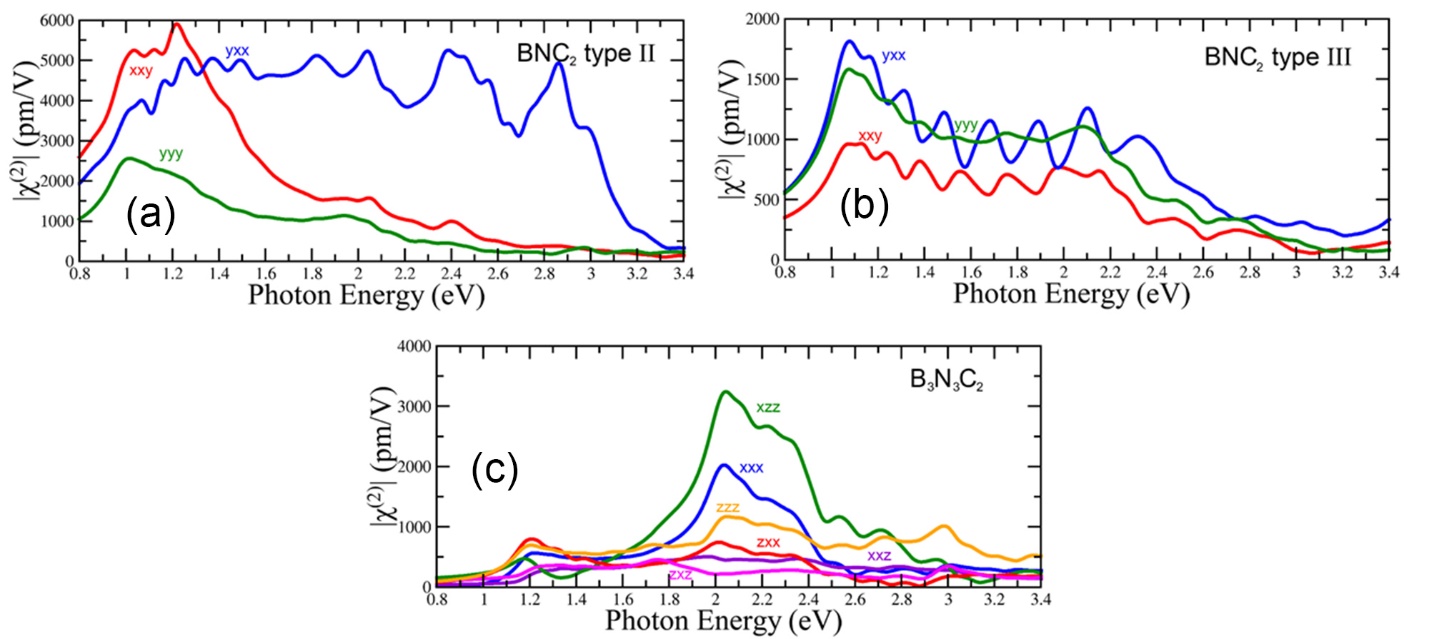


Figure S6. |χ^(2)^| components of the SHG tensor for BNC monolayer alloys: (a) BNC_2_ (type II); (b) BNC_2_ (type III); (c) B_3_N_3_C_2_.


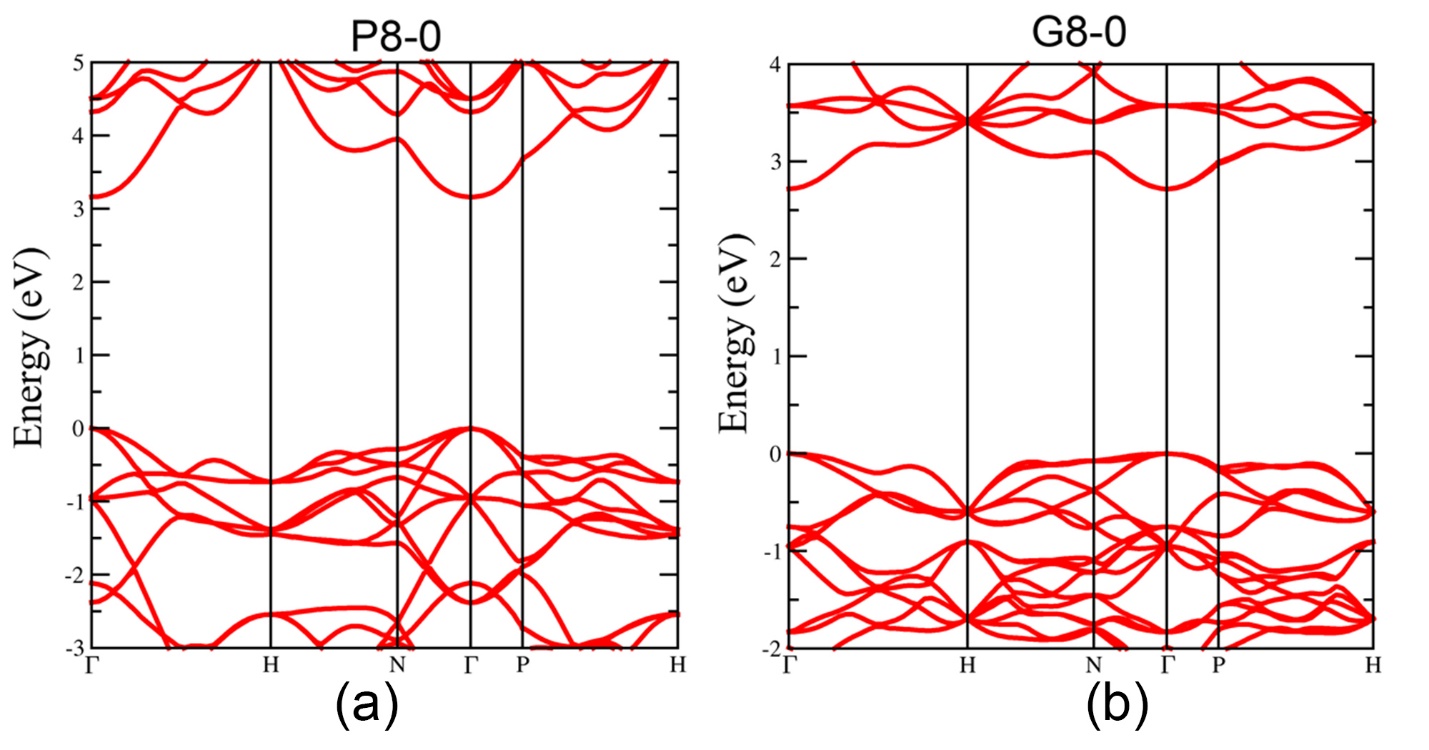


Figure S7. Band Structure of the BN Schwarzites: (a) P8-0 Schwarzite. (b) G8-0 Schwarzite. Valence band maximum is set to 0 eV.
